# Supplementary material for: Cryptococcus neoformans melanization incorporates multiple catecholamines to produce polytypic melanin
Source: J Biol Chem. 2021 Dec 20;298(1):101519. doi: 10.1016/j.jbc.2021.101519 (PMC8760516; doi:10.1016/j.jbc.2021.101519)
Supplement: Supplemental Figure S2 [file mmc2.pdf]

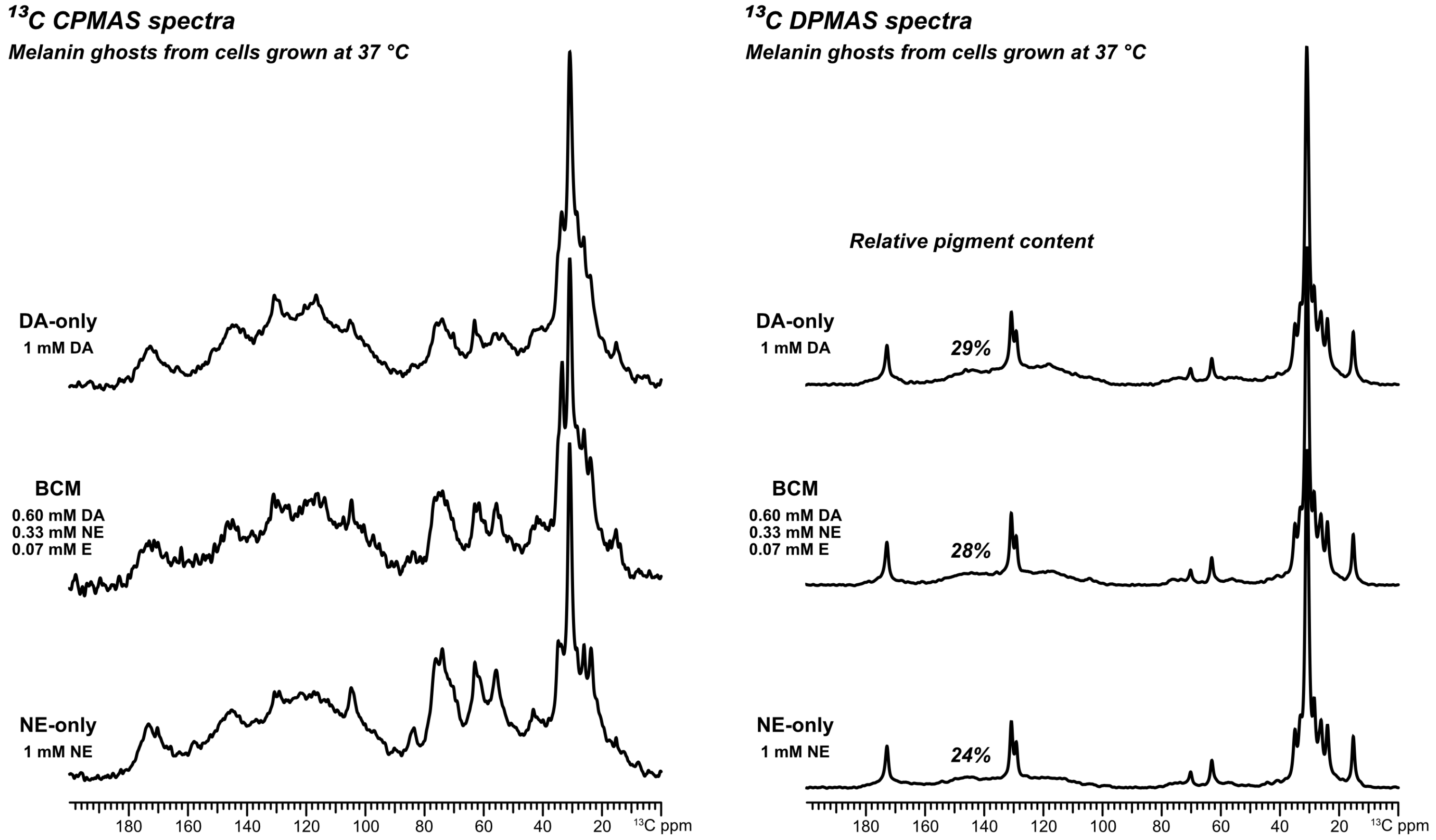

Figure S2. Solid-state NMR spectra of melanin 'ghosts' from *C. neoformans* cells grown with DA, NE, or BCM. 1D ^{13}C cross-polarization (CPMAS) spectra (left) and quantitatively-reliable 1D ^{13}C direct-polarization spectra (right) of melanin ghosts isolated from KN99α *C. neoformans* cultures grown for 16 days at 37˚C in minimal media containing either 1 mM dopamine (DA, top), 1 mM norepinephrine (NE, bottom) or in a 'brain catecholamine mixture' of 0.6 mM dopamine, 0.33 mM norepinephrine, and 0.07 mM epinephrine (BCM, middle). The DPMAS experiments were performed and the relative pigment content estimated as described in the legend of Figure S1.
